# Jatrorrhizine Improves Endothelial Function in Diabetes and Obesity through Suppression of Endoplasmic Reticulum Stress

**DOI:** 10.3390/ijms232012064

**Published:** 2022-10-11

**Authors:** Yan Zhou, Yuehan Wang, Chi Teng Vong, Yanyan Zhu, Baojun Xu, Cheng-Chao Ruan, Yitao Wang, Wai San Cheang

**Affiliations:** 1 State Key Laboratory of Quality Research in Chinese Medicine, Institute of Chinese Medical Sciences, University of Macau, Avenida da Universidade, Taipa, Macau 999078, China; 2 Food Science and Technology Program, BNU-HKBU United International College, Zhuhai 519087, China; 3 Department of Physiology and Pathophysiology, School of Basic Medical Sciences, Fudan University, Shanghai 200437, China

**Keywords:** diabetes, endothelial dysfunction, jatrorrhizine, endoplasmic reticulum stress, oxidative stress

## Abstract

Jatrorrhizine (JAT) is one of the major bioactive protoberberine alkaloids found in rhizoma coptidis, which has hypoglycemic and hypolipidemic potential. This study aimed to evaluate the vasoprotective effects of JAT in diabetes and obesity and the underlying mechanism involved. Mouse aortas, carotid arteries and human umbilical cord vein endothelial cells (HUVECs) were treated with risk factors (high glucose or tunicamycin) with and without JAT ex vivo and in vitro. Furthermore, aortas were obtained from mice with chronic treatment: (1) control; (2) diet-induced obese (DIO) mice fed a high-fat diet (45% kcal% fat) for 15 weeks; and (3) DIO mice orally administered JAT at 50 mg/kg/day for the last 5 weeks. High glucose or endoplasmic reticulum (ER) stress inducer tunicamycin impaired acetylcholine-induced endothelium-dependent relaxations (EDRs) in mouse aortas, induced oxidative stress in carotid arteries and HUVECs, downregulated phosphorylations of Akt at Ser473 and eNOS at Ser1177 and enhanced ER stress in mouse aortas and HUVECs, and these impairments were reversed by cotreatment with JAT. JAT increased NO release in high-glucose-treated mouse aortas and HUVECs. In addition, chronic JAT treatment restored endothelial function with EDRs comparable to the control, increased Akt/eNOS phosphorylation, and attenuated ER stress and oxidative stress in aortas from DIO mice. Blood pressure, glucose sensitivity, fatty liver and its morphological change, as well as plasma levels of aspartate aminotransferase (AST) and alanine aminotransferase (ALT) and plasma lipid profile, were also normalized by JAT treatment. Collectively, our data may be the first to reveal the vasoprotective effect of JAT that ameliorates endothelial dysfunction in diabetes and obesity through enhancement of the Akt/eNOS pathway and NO bioavailability, as well as suppression of ER stress and oxidative stress.

## 1. Introduction

Cardiovascular disease (CVD) is the main cause of death worldwide, and diabetes and obesity are major risk factors [1]. Endothelial dysfunction plays a key role in the pathogenesis of CVD and often occurs in diabetes, obesity, atherosclerosis, hyperglycemia and other vascular diseases [2]. Endothelial dysfunction is positively correlated with vascular diseases, and thus, it is important to study for therapeutic implications [3]. Endothelial dysfunction is mainly reflected in the reduction of vasodilators such as nitric oxide (NO) bioavailability and increased levels of vasoconstrictive substances, such as endothelin-1 (ET-1), angiotensin II, prostaglandin H_2_, reactive oxygen species (ROS) and so on. [4]. Oxidative stress and endothelial nitric oxide synthase (eNOS) uncoupling are important indicators of endothelial cell function [5]. Activity of eNOS controls vascular NO bioavailability under physiological conditions, while under pathological conditions, reduced eNOS activity will promote ROS levels and thereby endothelial dysfunction [6]. Protein kinase B (Akt) is known as the main signal modulator in vascular homeostasis to stimulate eNOS, and the Akt/eNOS pathway is suppressed in the diabetic vasculature [7].

The endoplasmic reticulum (ER) is a vital organelle for the synthesis, folding and transport of various secreted and membrane proteins [8]. Once this function is disrupted, the ER is unable to fold and transport newly synthesized proteins, resulting in ER stress, which is associated with cellular dysfunction by activating various pathways in cells [8]. The signaling cascade triggers the activation of three typical ER-associated proteins: PKR-like endoplasmic reticulum kinase (PERK), activating transcription factor 6 (ATF6) and inositol requiring enzyme 1 (IRE1) [9]. These proteins are involved in complex downstream signaling pathways and cause oxidative stress, inflammation and apoptosis in vascular tissues [10,11]. Excessive nutrients such as high glucose [12], glucosamine [13] and oxidized low-density lipoprotein cholesterol (ox-LDL) [14] induce vascular inflammation and endothelial dysfunction. On the other hand, alleviation of ER stress ameliorates cardiovascular complications induced by metabolic syndrome [15,16]. Considering the severe consequences of these diseases, strategies to prevent and treat endothelial dysfunction are worth exploring.

**Figure 1:**
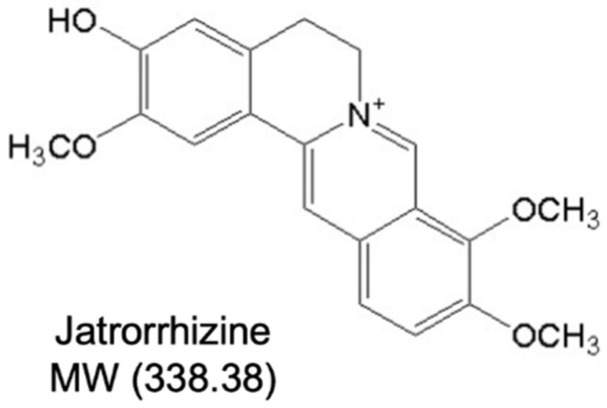
Structure of jatrorrhizine.

Compelling evidence has supported that Chinese herbal compounds or herbs are effective in preventing and treating diabetes mellitus and vascular dysfunction. Rhizoma coptidis (RC) is the dried rhizome of *Coptis chinensis* France that belongs to the Ranunculaceae family, exhibiting clinical applications for more than two thousand years and a wide range of pharmacological activities, including antibacterial, anti-inflammatory, anti-viral, anticancer, antiatherosclerotic, antiobesity and antidiabetes effects [17]. These properties are mainly attributed to protoberberine alkaloids in RC, including jatrorrhizine (JAT) (Figure 1), coptisine, berberine, epiberberine and palmatine. Previous studies have shown that berberine [18] and coptisine [19] improve endothelial function by inhibiting endoplasmic reticulum stress in hypertension and diabetes. However, only a few studies have reported the vascular activity of JAT. JAT possesses hypoglycemic [20] and hypolipidemic activities [21], but it remains unknown whether JAT protects against diabetes-associated endothelial dysfunction. This study aimed to investigate whether JAT has a protective effect against endothelial dysfunction in diabetes and obesity by modulating the Akt/eNOS pathway, ER stress, oxidative stress and NO bioavailability.

## 2. Results

### 2.1. Jatrorrhizine Improves Vascular Functions in Diabetes

**Figure 2:**
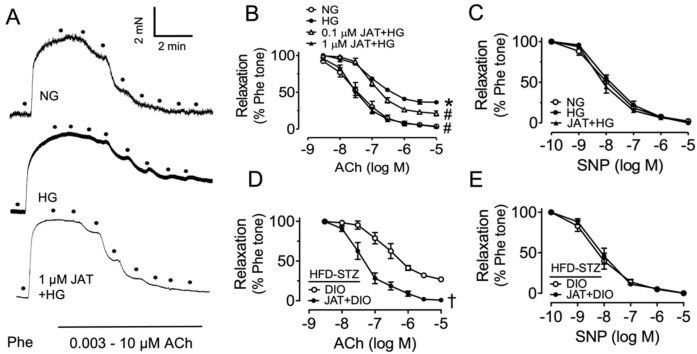
Vasoprotective effect of jatrorrhizine (JAT) in mouse aortas ex vivo. (**A**,**B**) Effect of JAT and high glucose (HG; 30 mM, 48 h) on the acetylcholine (ACh)-induced endothelium-dependent relaxations (EDRs) in aortas from C57BL/6J mice as compared with control (NG; mannitol added as osmotic control). (**C**) Sodium nitroprusside (SNP)-induced endothelium-independent relaxations were not affected. (**D**) Diet-induced obese (DIO) and diabetic mouse model was induced by high-fat diet (HFD; 45% kcal% fat, 10 weeks) plus single intraperitoneal injection of streptozotocin (STZ; 120 mg/kg) and ex vivo treatment of JAT (1 µM, 24 h) improved ACh-induced relaxations in aortas. (**E**) SNP-induced relaxations were unaffected. Results are mean ± SEM of 4 experiments. * *p* < 0.05 vs. NG; # *p* < 0.05 vs. HG; † *p* < 0.05 vs. DIO.

To evaluate the vasoprotective effect of jatrorrhizine (JAT) in mouse aortas ex vivo, we examined acetylcholine (ACh)-induced EDRs. After precontraction of endothelium-intact rings by phenylephrine (Phe, 3 μM) stimulation, ACh (3 nM–10 μM) was added to induce EDRs in the normal glucose control group (NG, mannitol added as osmotic control). Exposure to high glucose (30 mM, 48 h) mimicked diabetic hyperglycemia in mouse aortas, and we found that high-glucose induction impaired ACh-induced EDRs compared with control, which was significantly reversed by JAT (48 h) in a concentration-dependent manner (Figure 2A,B, Table 1). The effect of JAT at 1 μM was more effective than that at 0.1 μM, indicating that JAT at 1 μM exhibited more potent vascular protection. Endothelium-independent relaxations were measured in response to sodium nitroprusside (SNP, 1 nM–10 μM), reflecting that the response of vascular smooth muscle to NO was not affected (Figure 2C). These results show that JAT was effective in attenuating endothelial dysfunction in hyperglycemic conditions. The response to NO by smooth muscle in aortas was not altered.

We also studied the effect of JAT on a diabetes model to further demonstrate the vasoprotection of JAT. Mice were fed a high-fat diet (HFD, 45% kcal% fat for 10 weeks) plus a single intraperitoneal injection of streptozotocin (STZ, 120 mg/kg) to induce diabetes, which was confirmed by determination of 6 h fasting blood glucose >11 mM. Aortic segments isolated from diabetic mice were treated with JAT (1 µM) for 24 h. The impaired EDRs in diabetic mice were enhanced by JAT ex vivo (Figure 2D) without affecting SNP-induced vasodilatations (Figure 2E).

### 2.2. Jatrorrhizine Increases Akt/eNOS Phosphorylation and Alleviates ER Stress

**Figure 3:**
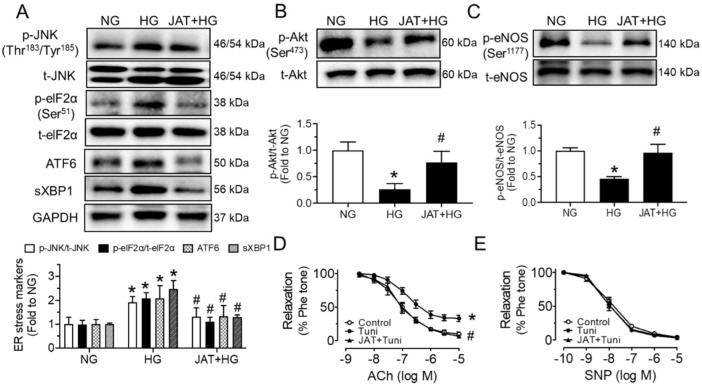
Jatrorrhizine attenuates endoplasmic reticulum (ER) stress and stimulates Akt/eNOS pathway in mouse aortas in hyperglycemic conditions ex vivo. (**A**–**C**) Western blotting data showing the expressions of ER stress markers, including phosphorylation and total JNK and elF2α, cleaved ATF6 and spliced XBP1; phosphorylation of eNOS at Ser^1177^ and total eNOS; and phosphorylation of Akt at Ser^473^ and total Akt in mouse aortas treated with HG (30 mM) and JAT (1 µM) for 48 h ex vivo. (**D**,**E**) JAT (1 µM) improved ACh-induced EDRs in mouse aortas treated with ER stress inducer tunicamycin (Tuni; 2 µg/mL, 24 h) ex vivo; with no effect on SNP-induced relaxations. Results are mean ± SEM of 4–5 experiments. * *p* < 0.05 vs. NG or Control; # *p* < 0.05 vs. HG or Tuni.

To estimate whether JAT restores endothelial function through the increase in Akt/eNOS phosphorylation and attenuation of ER stress, we performed Western blotting analyses and isometric force measurement. High glucose (30 mM, 48 h) induced the phosphorylation of JNK at Thr^183^/Tyr^185^ and eIF2α at Ser^51^, cleaved ATF6 and spliced XBP1 (sXBP1) in mouse aortas ex vivo, while these inductions were significantly alleviated by cotreatment with JAT at 1 µM for 48 h (Figure 3A). The upregulated expressions of the phospho-Akt at Ser^473^ and phospho-eNOS at Ser^1177^ in high-glucose-stimulated aortas were also reduced significantly by JAT treatment (1 µM), whereas the total protein levels of Akt and eNOS were not altered among the different treatment groups (Figure 3B,C). Furthermore, the EDRs of mouse aortas were directly impaired by ER stress inducer tunicamycin (Tuni; 2 µg/mL, 24 h), which were effectively improved by coincubation of JAT (1 µM, 24 h) without affecting SNP-induced relaxations (Figure 3D,E).

**Figure 4:**
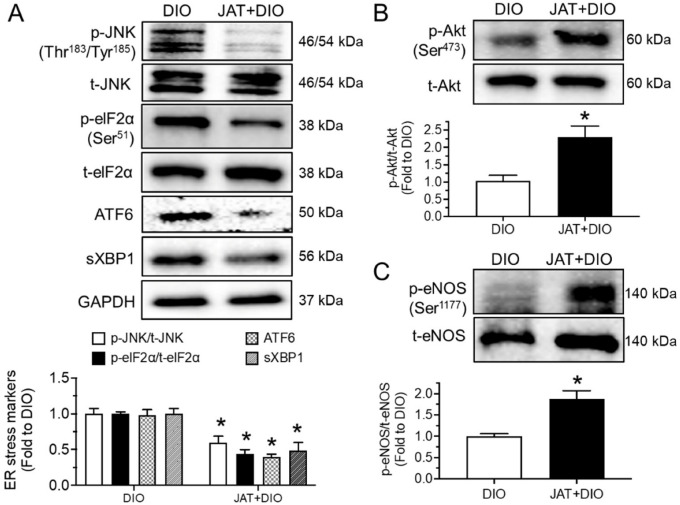
Effect of jatrorrhizine on endoplasmic reticulum stress and Akt/eNOS pathway in aortas from DIO mice ex vivo. (**A**–**C**) Western blotting of ER stress markers, phosphorylation and total Akt and eNOS in aortas from DIO mice induced by high-fat diet (45% kcal% fat) plus single intraperitoneal injection of streptozotocin (120 mg/kg) and treated with JAT (1 µM) for 24 h ex vivo. Results are mean ± SEM of 5–6 experiments. * *p* < 0.05 vs. DIO.

**Figure 5:**
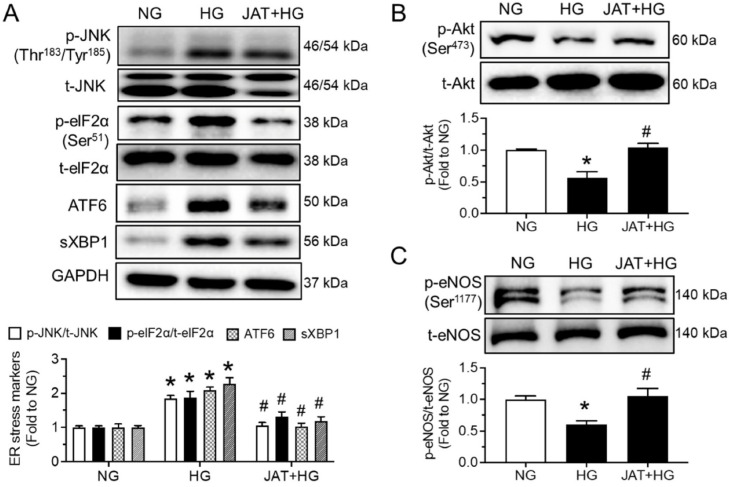
Effect of jatrorrhizine on endoplasmic reticulum stress and Akt/eNOS pathway in human umbilical cord vein endothelial cells (HUVECs). (**A**–**C**) Western blotting of ER stress markers and Akt/eNOS in HUVECs treated with HG (30 mM) and JAT (1 µM) for 48 h. Results are mean ± SEM of 5–6 experiments. * *p* < 0.05 vs. NG; # *p* < 0.05 vs. HG.

In line with the results in high-glucose-stimulated aortas, JAT (1 µM, 24 h) was found to effectively inhibit ER stress and the enhance Akt/eNOS pathway in the aortas of high-fat diet (45% kcal% fat)/streptozotocin-induced mice ex vivo (Figure 4A–C). Moreover, phosphorylation of Akt and eNOS was reduced, while ER stress markers were increased upon high-glucose exposure (30 mM, 48 h) in human umbilical cord vein endothelial cells (HUVECs) compared with protein levels in the control group (mannitol added as osmotic control). These changes were prevented by JAT at 1 µM for 48 h (Figure 5A–C).

### 2.3. Jatrorrhizine Suppresses Oxidative Stress and Increases NO Bioavailability

**Figure 6:**
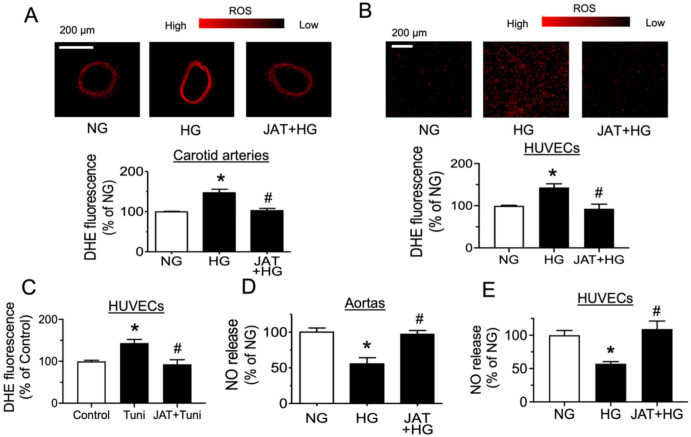
Jatrorrhizine suppresses oxidative stress and increases nitric oxide (NO) bioavailability. (**A**,**B**) Representative images and summarized data showing exposure to high glucose (30 mM, 48 h) increased the level of reactive oxygen species (ROS) in mouse carotid arteries and HUVECs, and such elevation was decreased by JAT at 1 µM as measured by dihydroethidium (DHE) staining. (**C**) JAT (1 µM) treatment inhibited the tunicamycin (tuni; 2 µg/mL, 1 h)-triggered ROS generation in HUVECs. (**D**,**E**) NO release from mouse aortas and HUVECs upon high glucose (30 mM) stimulation and cotreatment with JAT (1 µM) for 48 h as assessed by measuring the nitrite level in culture medium using Griess reagent. Results are mean ± SEM of 4-6 experiments. * *p* < 0.05 vs. NG or Control; # *p* < 0.05 vs. HG or Tuni.

The role of oxidative stress and NO bioavailability in high-glucose-induced endothelial dysfunction was further investigated by fluorescence imaging and Griess assay. The results show that ROS level was elevated in mouse carotid arteries and HUVECs after treating with high glucose (30 mM), and this increase was decreased by JAT at 1 µM for 48 h as measured by dihydroethidium (DHE) staining (Figure 6A,B). In addition, incubation with tunicamycin (2 µg/mL, 1 h) increased ROS level in HUVECs, implying that ER stress can lead to oxidative stress. JAT (1 µM, 1 h) and remarkably inhibited tunicamycin-triggered ROS generation (Figure 6C). The results of nitrite level in culture medium by Griess reagent demonstrated that NO release was diminished by high glucose at 30 mM in mouse aortas and HUVECs, whereas NO production was greatly improved by JAT at 1 µM for 48 h (Figure 6D,E).

### 2.4. Chronic Jatrorrhizine Treatment Attenuates Endothelial Function in Diabetic and Obese Mice

**Figure 7:**
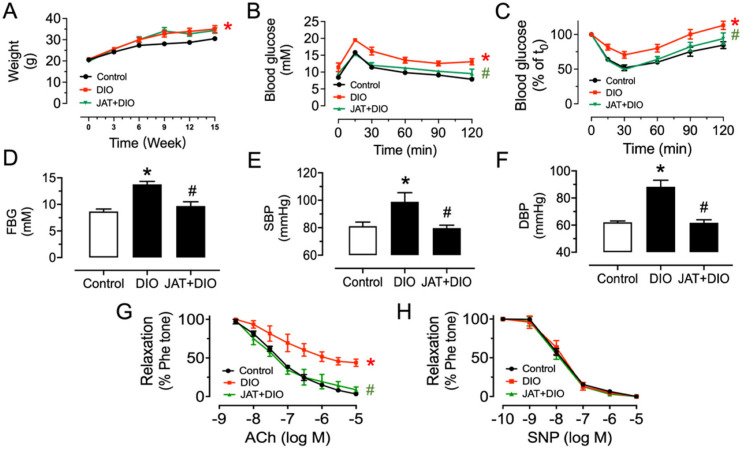
Jatrorrhizine treatment improves blood glucose, blood pressure and vascular relaxations in DIO mice. (**A**) Body weight in mice fed a high-fat diet or normal chow for 15 weeks and oral administered with JAT at 50 mg/kg body weight daily for the last 5 weeks. (**B**) Oral glucose tolerance test (OGTT) upon 6 h fasting. (**C**) Insulin tolerance test (ITT) upon 2 h fasting. (**D**) Fasting blood glucose (FBG) upon 6 h fasting. (**E**,**F**) Changes in systolic (SBP) and diastolic (DBP) blood pressure measured by tail-cuff method. (**G**) Effect of oral administration of JAT (50 mg/kg/day, 5 weeks) on ACh-induced relaxations in aortas from DIO mice. (**H**) SNP-induced endothelium-independent relaxations. Data are mean ± SEM from six mice for each group. * *p* < 0.05 vs. Control; # *p* < 0.05 vs. DIO.

Based on the results from ex vivo and in vitro experiments, we examined the vascular benefit of chronic JAT treatment in diabetic and obese mice. High-fat diet feeding for 15 weeks significantly increased body weight as compared with control mice, but chronic administration with JAT did not reduce body weight (Figure 7A). The type 2 diabetic mouse model was successfully established as supported by impaired glucose tolerance and insulin sensitivity, as well as elevated fasting blood glucose (>11 mM) (Figure 7B–D). JAT was effective in normalizing glucose tolerance, insulin sensitivity and fasting blood glucose level. JAT treatment also reduced systolic and diastolic blood pressures in DIO mice (Figure 7E,F). JAT significantly improved ACh-induced EDRs without affecting SNP-induced endothelium-independent relaxations in the aortas of diabetic and obese mice (Figure 7G,H).

### 2.5. Jatrorrhizine Improves Liver and Plasma Lipid Profile in DIO Mice

**Figure 8:**
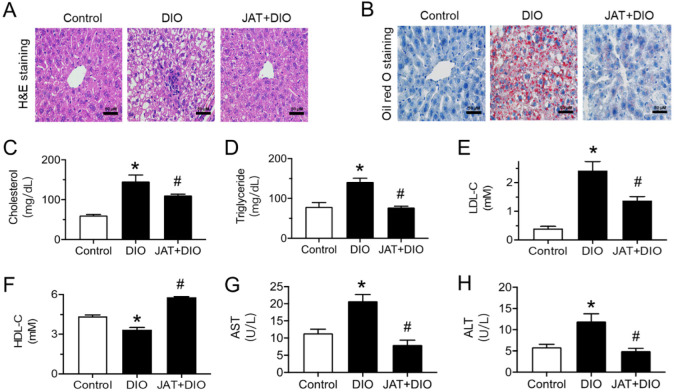
Effect of jatrorrhizine on liver and plasma lipid profile in DIO mice. (**A**,**B**) Representative images of H&E staining and oil red O staining sections of livers from mice (scale bar: 50 µm). (**C**–**H**) Plasma levels of total cholesterol, triglyceride, low-density lipoprotein cholesterol (LDL-C), high-density lipoprotein cholesterol (HDL-C), aspartate aminotransferase (AST) and alanine aminotransferase (ALT) measured by corresponding test kits. Data are mean ± SEM from six mice for each group. * *p* < 0.05 vs. Control; # *p* < 005 vs. DIO.

To examine whether JAT treatment affected hepatic lipid accumulation, we investigated histopathological changes in the liver. H&E staining showed that the structure of hepatocytes changed to cloudy swelling with vacuolization of the cytoplasm, and the cells were loosely arranged in the livers of vehicle-treated DIO mice, while these histopathological changes in liver tissues were restored by JAT treatment (Figure 8A). Oil red O staining confirmed that oral administration of JAT significantly reduced lipid droplets in the livers as compared with the DIO mice group (Figure 8B). These results indicate that JAT treatment effectively ameliorated hepatic lipid accumulation.

In addition, the basic metabolic parameters of DIO mice in the control, DIO and JAT groups were detected to further explore the effect of JAT on lipid profile and liver protection. The results of the plasma lipid profile demonstrated that JAT treatment reduced cholesterol and triglyceride content in DIO mice (Figure 8C,D). Low-density lipoprotein cholesterol (LDL-C) level was elevated, and high-density lipoprotein cholesterol (HDL-C) level was reduced in plasma from DIO mice, which were reversed by chronic JAT treatment (Figure 8E,F). Furthermore, JAT administration decreased the plasma levels of aspartate aminotransferase (AST) and alanine aminotransferase (ALT) which were indicators for liver damage (Figure 8G,H).

### 2.6. Jatrorrhizine Treatment Activates Akt/eNOS Pathway and Suppresses ER Stress and Oxidative Stress in Aortas of DIO Mice

**Figure 9:**
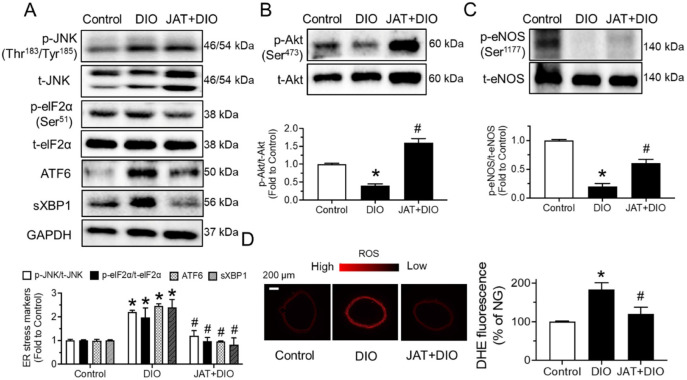
Chronic jatrorrhizine treatment attenuates endoplasmic reticulum stress and increases Akt/eNOS pathway in DIO mice. (**A**–**C**) The expressions of ER stress markers (JNK, eIF2α, cleaved ATF6 and spliced XBP1) and Akt/eNOS in aortas from DIO mice. (**D**) Changes in ROS as measured by dihydroethidium (DHE) staining. Data are mean ± SEM from 5–6 mice for each group. * *p* < 0.05 vs. Control; # *p* < 0.05 vs. DIO.

To find whether chronic JAT treatment impacts ER stress and oxidative stress, Western blotting and dihydroethidium (DHE) staining were conducted. We found that the expression levels of ER stress markers, including phosphorylation of JNK at Thr^183^/Tyr^185^ and eIF2α at Ser^51^, cleaved ATF6 and spliced XBP1, were significantly higher in aortas from DIO mice than in the control, whereas JAT treatment reversed these changes (Figure 9A). JAT elevated Akt and eNOS phosphorylation in DIO mice (Figure 9B,C). Additionally, the enhanced ROS level was effectively inhibited by JAT treatment in mouse aortas (Figure 9D).

## 3. Discussion

Our current findings support the therapeutic value of JAT for ameliorating diabetes-related endothelial dysfunction by upregulating the Akt/eNOS pathway to increase NO bioavailability, accomplished with inhibition of ER stress and oxidative stress in vitro and in vivo.

First, hyperglycemia and dyslipidemia contribute to endothelial dysfunction and thus hypertension in patients with metabolic syndrome [22]. A high glucose level decreases NO bioavailability and increases generation of ROS, impairing vasorelaxations [23]. Endothelial nitric oxide synthase (eNOS) is responsible for catalyzing the oxidation of L-arginine to form NO and L-citrulline in the presence of cofactors such as tetrahydrobiopterin [24]. The activity and/or expression of eNOS are particularly important to regulate endothelial function. Akt is one of the various modulators controlling eNOS activity. In diabetes, the Akt/eNOS signaling pathway is downregulated in the vasculature, and strategies to activate Akt/eNOS are effective in ameliorating vascular dysfunction [25].

Of note, ER stress is demonstrated to be closely associated with the pathogenesis of vascular diseases such as atherosclerosis, obesity and type 2 diabetes [26]. Vascular risk factors such as high glucose [16], oxLDL [27] and homocysteine [28] are known to trigger ER stress in endothelial cells. Upon induction of ER stress, Akt/eNOS activity is reduced, resulting in a decrease in NO production and an increase in ROS level, thereby causing endothelial injury [29,30]. In agreement with previous studies, we found that stimulation of high glucose attenuated endothelium-dependent relaxations in aortas ex vivo, downregulated Akt/eNOS phosphorylation and upregulated ER stress markers in aortas and HUVECs, elevated ROS generation in carotid arteries and HUVECs, and reduced NO level in culture medium. Such impairments were reversed by coincubation with JAT. Moreover, our ex vivo experiments using tunicamycin confirmed that alleviation of ER stress can directly lead to impaired endothelium-dependent relaxations and enhanced oxidative stress in endothelial cells. The present study is the first to demonstrate that JAT can relieve ER stress and subsequently restore endothelial function. The SNP-induced endothelium-dependent relaxations were similar among different groups, implying that the response of vascular smooth muscle to NO was unaffected and that the impairment of ACh-induced relaxations is attributed to the lowered NO production from endothelial cells. Our in vitro experiments demonstrated that coincubation of JAT greatly increased NO bioavailability in high-glucose treatment and improved endothelial cell function.

ER stress, oxidative stress and NO bioavailability are closely linked events in modulating vascular homeostasis [10,11]. ER function is disrupted for various reasons, resulting in the accumulation of misfolded or unfolded proteins, which causes ER stress. Oxidative stress plays a critical role in endothelial function, occurring due to the unbalanced production of both ROS and antioxidants. NADPH oxidase is considered a major source of ROS and can be upregulated and activated in response to risk factors. The excessive production of ROS leads to the accumulation of toxic products, which affect the normal function of cells and lead to cell death [31]. There is much evidence that protein misfolding associated with cardiometabolic diseases stimulates the overproduction of ROS in the vascular wall [32]. Existing evidence has also suggested that ER stress can stimulate oxidative stress by disrupting the antioxidant defense mechanism [11]. Likewise, we observed that tunicamycin as an ER stress inducer increased ROS levels in HUVECs, indicating that ER stress can indeed cause oxidative stress. Moreover, many cardiovascular risk factors such as diabetes, obesity and hypertension reduce NO bioavailability through NADPH oxidase and uncoupling of eNOS, resulting in the production of superoxide anion [33]. Decreased NO production leads to vasoconstriction, platelet aggregation and endothelial dysfunction.

Chronic administration of JAT confers vasoprotection in diabetes and obesity in vivo, consistent with the ex vivo results in aortas exposed to high glucose or tunicamycin. The current findings suggest that JAT treatment for 5 weeks is highly effective in restoring endothelial function and mitigating hypertension in obese and diabetic mice. As illustrated by Western blot protein bands and fluorescent images, the decrease in both ER stress and oxidative stress, as well as upregulation of Akt/eNOS and NO bioavailability, contributed to the improvement in endothelial function. Among the various active ingredients of RC, berberine is recognized as an effective hypoglycemic drug for diabetes and has been shown to improve endothelial dysfunction by reducing ER stress [34]. Notably, the hypoglycemic ability of JAT is significantly higher than that of berberine [35]. Comparisons of the vasoprotective activities with berberine and other ingredients of RC remain to be evaluated in the future to identify potent candidates. The in vivo results are in line with previous studies. Chronic intake of JAT exerted beneficial effects on glucose tolerance, insulin sensitivity, lipid profile and liver function.

Metabolic syndromes involving hypertension, diabetes and obesity have been reported to induce oxidized and glycated LDL cholesterol, free fatty acids and specific aminotransferases that contribute to endothelial dysfunction and other vascular diseases [36,37]. In our in vivo experiments in mice, JAT treatment remarkably reduced hepatic fat accumulation and morphological deterioration. Liver damage was prevented by JAT as supported by the suppressed levels of ALT and AST in plasma from DIO mice. Moreover, JAT ameliorated dyslipidemia, diminishing plasma levels of total cholesterol, triglyceride and LDL while augmenting HDL levels. These ameliorations in glucose and lipid metabolism might partially contribute to the protective effect of JAT on endothelial function; nevertheless, a direct vascular protective property of JAT is strongly supported in ex vivo experiments where the ambient glucose and lipid levels were constant.

## 4. Materials and Methods

### 4.1. Animal Experiments

All animal care and research protocols were approved by the Animal Research Ethical Committee of the University of Macau (Macao, China) and in accordance with the National Institutes of Health guidelines for the Care Use of Laboratory Animals. Male C57BL/6J mice (6–8 weeks old) were obtained from the Faculty of the Healthy Science Animal Centre of the University of Macau and housed in animal holding rooms with controlled temperature (22 ± 1 °C) with 12 h light/dark cycles. Mice were randomly divided into three groups for chronic treatment: (1) control fed a normal chow diet for 15 weeks and orally administered water (vehicle) for the last 5 weeks; (2) diet-induced obese (DIO) mice fed a high-fat diet (45% kcal% fat) for 15 weeks with 5-week vehicle treatment; and (3) DIO mice orally administered JAT at 50 mg/kg/day for the last 5 weeks (purity > 97%; Shanghai Aladdin Bio-Chem Technology Co. Ltd., Shanghai, China). A commercial blood glucose meter was used to measure fasting blood glucose (6 h fasting), and mice with fasting blood glucose of >11 mM were considered type 2 diabetic mice. Food intake and body weight of mice were recorded every 3 weeks.

### 4.2. Blood Pressure Measurement

After 6 h of fasting, systolic (SBP) and diastolic (DBP) blood pressures were detected using the tail-cuff method (CODA noninvasive blood pressure system; Kent Scientific Corporation, Torrington, CT, USA) in conscious mice.

### 4.3. Blood Glucose Measurement

After 6 h of fasting, blood was collected from the tail of mice. Fasting blood glucose (FBG) level was measured by a commercial glucometer (Jiangsu Yuyue Medical Equipment and Supply Co. Ltd., Jiangsu, China), and mice with FBG >11 mM were considered diabetic mice. For the oral glucose tolerance test (OGTT), after fasting for 6 h, mice were administered glucose solution (1.2 g/kg body weight) by intragastric administration, and blood glucose levels were detected at 0, 15, 30, 60, 90 and 120 min. For the insulin tolerance test (ITT), after fasting for 2 h, mice were administered insulin (0.5 U/kg body weight) by intraperitoneal injection, and blood glucose levels were measured at the same time intervals as OGTT.

### 4.4. Determination of Plasma Lipid Profile

Mice were sacrificed by CO_2_ inhalation, and plasma was collected to detect total cholesterol (TC), triglyceride (TG; Stanbio Laboratory, Boerne, TX, USA), high-density lipoprotein cholesterol (HDL-C) and low-density lipoprotein cholesterol (LDL-C) (Nanjing Jiancheng Bioengineering Institute, Nanjing, China) levels by corresponding commercial test kits. Subsequently, plasma alanine aminotransferase (ALT) and aspartate aminotransferase (AST) were analyzed colorimetrically utilizing assay test kits (Nanjing Jiancheng Bioengineering Institute, Nanjing, China).

### 4.5. Hematoxylin and Eosin Staining and Oil Red O Staining

To observe histopathologic alterations, the liver tissue at the same location of the left liver was obtained from each mouse to be fixed in 10% buffered formalin, embedded in paraffin, sliced in 3–5 μm thick sections and stained with hematoxylin and eosin (H&E) (Beyotime Biotechnology, Shanghai, China). Liver sections were also fixed in 3.7% formaldehyde, immersed in oil red O solution to observe the lipid droplets and stained with hematoxylin to visualize nuclei (Beyotime Biotechnology, Shanghai, China). H&E samples and oil red O slides were examined using a microscope and photographed under ×400 magnification.

### 4.6. Culture of Human Umbilical Cord Vein Endothelial Cells (HUVECs)

Human umbilical cord vein endothelial cells (HUVECs) obtained from Lonza were grown in F-12K medium (7 mM glucose present in medium) supplemented with 30 μg/mL endothelial cell growth supplement (ECGS, Sigma-Aldrich, St. Louis, MO, USA) and 100 μg/mL heparin (Alfa Aesar, Stoughton, MA, USA). Cells were cultured in 75 cm^2^ flasks, 96-well plates, 24-well plates and 6-well plates precoated with 0.2% gelatin and maintained at 37 °C in a humidified atmosphere of 5% CO_2_. Medium was changed every two days and passaged by 0.5% trypsin-EDTA (Gibco, Grand Island, NY, USA). HUVECs at 4–8 passages were used for experiments. HUVECs were treated with mannitol as osmotic control, high glucose (30 mM, 48 h; Sigma-Aldrich, St. Louis, MO, USA) and cotreated with JAT (1 µM).

### 4.7. Ex Vivo Culture of Mouse Aortas

After mice were sacrificed by CO_2_ inhalation, mouse thoracic aortas and carotid arteries were dissected and cleaned in sterile phosphate-buffered saline (PBS) and then cut into segments (~2 mm) and incubated in Dulbecco’s modified Eagle’s medium (5.55 mM glucose present in DMEM, Gibco, Grand Island, NY, USA) supplemented with 10% fetal bovine serum (FBS, Gibco, Paisley, UK) and 1% penicillin/streptomycin (P/S, Gibco, Grand Island, NY, USA). Mannitol as osmotic control, high glucose (HG; 30 mM, 48 h, Sigma-Aldrich, ST. Louis, MO, USA), ER stress inducer tunicamycin (Tuni; 2 µg/mL, 24 h) and JAT (0.1 or 1 µM; Shanghai Aladdin Bio-Chem 96 Technology Co. Ltd., Shanghai, China) were added individually into the culture medium that bathed the aortic rings in an incubator of 5% CO_2_ at 37 °C. After the incubation period, segments were transferred to fresh Krebs solution for functional studies in a wire myograph and were frozen for Western blotting and fluorescence imaging.

### 4.8. Isometric Force Measurement in Wire Myograph

After mice were sacrificed, segments of mouse aortas (~2 mm) cultured for 24 or 48 h in DMEM and aortas (~2 mm) dissected directly after modeling were mounted on a Multi Myograph System (Danish Myo Technology, Aarhus, Denmark) with 5 mL Krebs–Henseleit solution, and changes in isometric tension were measured. Each experiment was performed on rings obtained from different mice. Mouse aortas were initially stretched to an optimal baseline tension of 3 mN and were then equilibrated for 60 min before the start of the experiments at 37 °C. Each ring was contracted by 60 mM KCl and rinsed five times in Krebs solution. Subsequently, to examine endothelium-dependent relaxations (EDRs) and endothelium-independent relaxations, each ring was contracted with phenylephrine (Phe, 3 μM, α1-adrenoceptor agonist, Sigma-Aldrich, St. Louis, MO, USA) to produce a sustained contraction and then was relaxed by cumulative addition of acetylcholine (ACh, 3 nM—10 µM, muscarinic acetylcholine receptor agonist, Sigma-Aldrich, St. Louis, MO, USA) and measured in response to sodium nitroprusside (SNP, 1 nM—10 µM, exogenous NO donor, Sigma-Aldrich, St. Louis, MO, USA).

### 4.9. Western Blotting Assay

Total protein was isolated from the frozen aortas in liquid nitrogen or HUVECs after treatment with ice-cold RIPA lysis buffer. The supernatant after centrifugation was collected and measured for protein concentration by bicinchoninic acid (BCA) assay (Beyotime Institute of Biotechnology, Shanghai, China). Proteins in 20 µg quantities were separated by electrophoresis on 10% sodium dodecyl sulfate polyacrylamide gel electrophoresis (SDS-PAGE) and transferred onto a polyvinylidene difluoride (PVDF) membrane (Millipore, Billerica, MA, USA). After blocking with 1% BSA in 1× TBST for 2 h at room temperature, the membranes were washed and then probed with specific primary antibodies against phosphor (p)-Akt at Ser^473^ (No. 4060S), Akt (No. 4685S), p-eNOS at Ser^1177^ (No. 9570S), eNOS (No. 32027S), p-JNK at Thr^183^/Tyr^185^ (No. 9255S), JNK (No. 9253S), p-eIF2α at Ser^51^ (No. 3398S) (1:1000 dilution; Cell Signaling Technology, Danvers, MA), eIF2α (1:1000 dilution; No. 11170; Proteintech Group, Inc., Wuhan, China), Anti-XBP1 (1:1000 dilution; No. ab220783), ATF6 (1:1000 dilution; No. ab203119) (Abcam, Cambridge, UK), and GAPDH (1:5000 dilution; No. 60004S; Proteintech Group, Inc., Wuhan, China) at 4 °C overnight and secondary antibodies (1:1000 dilution; Beyotime, Shanghai, China). Protein bands were detected with the American ECLTM Advanced Western Blotting Detection Kit (GE Healthcare Life Sciences, Uppsala, Sweden) and imaged with the ChemiDoc^TM^ MP Imaging System (Bio-Rad, Hercules, CA, USA).

### 4.10. Determination of ROS by Dihydroethidium (DHE) Staining

The intracellular ROS levels in HUVECs and cross-sectional areas of aortas were detected by DHE probe as in our previous study [19]. Segments of mouse thoracic aortas (ex vivo treatment or in vivo treatment) and carotid arteries (ex vivo treatment) were embedded in OCT compound (Tissue-Tek, Sakura, Nagano, Japan) and cut into sections of 10 μm thickness using a Leica CM 1000 cryostat at −20 °C. Frozen sections of aortas and carotid arteries or cultured HUVECs were washed with PBS and incubated for 15 min with DHE (5 μM)-containing normal physiological saline solution (NPSS) at 37 °C in the dark. After the samples were washed with PBS to remove the dye, fluorescence images were observed by the Leica TCS SP8 Confocal Laser Scanning Microscope System (Leica Microsystems, Germany; 515 nm excitation; 585 nm emission). DHE fluorescence intensity was measured by ImageJ 1.53a software.

### 4.11. Determination of NO Generation

Aortic segments or HUVECs were treated with high glucose (HG; 30 mM) and JAT (1 µM) in a 24-well plate for 48 h, and the supernatants were collected to detect the amount of NO by Griess reagent (Invitrogen, Oregon, USA) according to the manufacturer’s instructions. The SpectraMax M5 microplate reader (Molecular Devices, Silicon Valley, CA, USA) was used to read absorbance at 548 nm.

### 4.12. Statistical Analysis

All data were expressed as mean ± standard error of mean (SEM) of *n* separate experiments. There were six mice for each group in animal experiments. Relaxations were expressed as percentage reduction in phenylephrine-induced contraction. Dilator concentrations in negative logarithm inducing 50% of maximum response (*p*D_2_) and maximum relaxation (*E*_max_%) were calculated. Comparison was performed using GraphPad Prism 9.0 (version 8, GraphPad Software InC., San Diego, CA, USA) by Student’s *t*-test or one-way analysis of variance (ANOVA), followed by Bonferroni post hoc tests. Differences with *p* < 0.05 were considered statistically significant.

## 5. Conclusions

The present study uncovers both ex vivo and in vitro that coincubation with JAT ameliorates endothelial function to enhance vasodilatation, by increasing Akt/eNOS activity and NO bioavailability, as well as decreasing ER stress and oxidative stress, which are consistent with the beneficial effects of chronic administration of JAT in vivo. JAT treatment also normalizes metabolic profiles and blood pressure in diabetic and obese mice. These data provide new mechanistic insights to support the potential therapeutic use of JAT for combating vascular diseases associated with metabolic disorders.

## Figures and Tables

**Table 1:** Dilator concentrations in negative logarithm inducing 50% of maximum response (*p*D_2_) and maximum relaxation (*E*_max_%) for acetylcholine (ACh)-induced endothelium-dependent relaxations (EDRs) of mouse aortas treated with high glucose (HG; 30 mM, 48 h) and different concentrations of jatrorrhizine (JAT), with mannitol as control (NG). Results are mean ± SEM of 4 experiments. * *p* < 0.05 vs. NG; # *p* < 0.05 vs. HG.

Treatment	*p*D_2_	*E*_max_%
**NG**	7.48 ± 0.12	95.38 ± 2.96
**HG**	6.97 ± 0.05 *	65.05 ± 1.29 *
**0.1 µM JAT+HG**	6.97 ± 0.07	80.96 ± 2.08 #
**1 µM JAT+HG**	7.57 ± 0.06 #	95.72 ± 1.60 #

## Data Availability

The data presented in this study are available on request from the corresponding author.
